# Survival status and mortality predictors among severely malnourished under 5 years of age children admitted to Minia University maternity and children hospital

**DOI:** 10.1186/s12887-020-02146-1

**Published:** 2020-05-19

**Authors:** Eman Ramadan Ghazawy, Gihan Mohammed Bebars, Ehab Salah Eshak

**Affiliations:** 1 Public Health and Preventive Medicine department, Faculty of medicine, El-Minia University. University St, El-Minia, 1666 Egypt; 2 Pediatrics Department, Faculty of Medicine, Minia University, Minia, Egypt

**Keywords:** Severe malnutrition, Survival status, Predictors of mortality

## Abstract

**Background:**

Though effective treatment programs for severely malnourished children are available, mortality rate among children with acute malnutrition continue to rise and little is known about its long-term outcomes and potential predictors of its in-hospital and post-discharge mortality. The aim of this study was to assess the survival status and predictors for mortality in severely malnourished children admitted to Minia University Maternity and Children Hospital.

**Methods:**

A retrospective cohort study which included 135 children under 5 years of age who were admitted to the nutrition rehabilitation ward with severe acute malnutrition (SAM) during the period from January to December 2018. Data were collected from the inpatient’s hospital records and the children’s parents/guardians were interviewed using a detailed structured questionnaire that inquired about demographic and socioeconomic variables. The logistic and Cox regressions were used to assess the factors associated with the SAM’s mortality.

**Results:**

A total of 135 children were enrolled into the study. Death rate during hospitalization was 9.6%. The survival rate at the end of the fourth week of admission was 82.4%. There were 6.7% post-discharge deaths among 104 alive discharged children which occurred within 8 weeks after discharge. The adjusted HRs (95% CIs) for total SAM deaths were 1.57 (1.10–2.99) in children < 12 vs ≥ 12 months old; 4.79 (2.23–6.10) in those with WAZ < −3SD, 2.99 (1.16–4.66) in those with edema at admission and 3.44 (1.07–9.86) in children with complications. The respective ORs (95%CIs) for in-hospital SAM deaths in the same groups of children were 2.64 (1.22–6.43), 8.10 (2.16–11.67), 3.04 (1.70–6.06) and 3.71 (1.59–6.78). The main predictor for the SAM’s post-discharge mortality was illiteracy of mothers; the adjusted HR (95%CI) was 7.10 (1.58–31.93; *p* = 0.01).

**Conclusions:**

Age, WAZ, edema and complications at admission were predictors for both in-hospital and total SAM mortality, while mother’s education contributed to the early post-discharge mortality. The identification of predictors for mortality is an important preliminary step for interventions aiming to reduce morbidity and mortality.

## Background

Malnutrition is one of the most common causes of morbidity and mortality among children all over the world. Of the 7.6 million annual deaths among children who are under 5 years of age [1]. Approximately 35% are due to nutrition-related factors and 4.4% of deaths have been shown to be specifically attributable to severe wasting [2]. Despite the availability of outpatient treatment, the number of children with Sever Acute Malnutrition (SAM) seeking admission at hospitals is increasing [1, 2]. SAM is defined globally as a very low weight for length/height (WFL/WFH) below – 3 zscores of the median WHO growth standards, or less than 70% of the median National Center for Health Statistics standard or the presence of nutritional edema. In children aged 6–59 months, mid-upper arm circumference (MUAC) less than 11.5 cm is also an indicator of severe acute malnutrition [3]. Globally, 17 million children under 5 were affected by SAM [4]. In Egypt, there were no reports on accurate estimates for the prevalence of SAM. However, the anthropometric estimates from the Egypt Demography and Health Survey (EDHS) 2014 have indicated that 5.5% of the under-5 children were underweight, 8.4% were wasted and 21.4% were stunted [5]. A cross-sectional study in Alexandria has found under-weight, stunting and wasting in 7.3, 15 and 3.6% of the 1217 preschool children aged 6–71 months [6]; while the estimated proportions among 400 under-5 children in Fayoum were 23.2, 18.5 and 19.3%, respectively [7].

Unfortunately, more than one-fourth of SAM deaths occur during hospitalization [8]. Studies suggest that the possible causes for high mortality rate could be attributed to the severity of illness at presentation, comorbidities and faulty in management [9–11]. Additionally, a high rate of mortality in the months following hospital discharge has been observed among children with SAM in sub-Saharan Africa [12–17], a recent systematic review reported paediatric post-discharge mortality rates in resource-poor countries of up to 18% which may exceed in-hospital mortality rates in many settings. This implies that processes underlying susceptibility to mortality continue beyond the clinically evident acute episode. In these studies, poor nutritional status, young age, and HIV were all associated with higher mortality risk post-discharge [15, 18].

Studying the treatment’s outcomes of malnutrition and the potential predictors of mortality among severely malnourished children admitted to hospitals is a crucial step for enhancing the quality of care provided to malnourished children. However, there is a paucity of studies that reported mortality outcomes in severely malnourished children in general and especially after being discharged from inpatient facilities [18–20], and none of the available studies was conducted in Egypt. Therefore, the objective of this study was to assess the survival status and predictors for the mortality (total, in-hospital and post-discharge) in severely malnourished under 5 years children admitted to Minia University Maternity and Children Hospital.

## Methods

### Study setting and population

This retrospective cohort study was conducted at Minia University Maternity and Children Hospital, the only referral and teaching hospital in Minia governorate, Egypt. This hospital provides a wide range of health care services for urban and rural populations from near and far districts in Minia Governorate. The hospital has a Nutrition Rehabilitation Unit with a capacity of 60 beds and serves as a treatment center for children with malnutrition based on the standardized WHO protocol. The average number of examined malnourished children in this unit is around 40 patients per week and the unit annually serves, on average, 150 inpatient and 2000 outpatient malnourished children.

All children under 5 years of age who were admitted with SAM to the nutrition rehabilitation ward during the period from January to December 2018 were recruited for this study. SAM was diagnosed by the presence of severe wasting [z score for weight for height (WHZ) < − 3.0 SD and/or the presence of nutritional edema [3]. All admitted children with SAM were managed according to the WHO protocol for management of SAM [3] and passed through initial stabilization phase (with the use of F75) and rehabilitation phase (F100). Whenever needed, other lines of treatment were also provided according to the WHO guidelines updates [3] after performing the required investigations such as stool analysis, complete blood picture, levels of C-reactive protein, blood glucose level, serum electrolytes (Na, K and ionized Ca), renal function tests and liver function tests. A daily check of weight gain during the treatment course was conducted and any comorbidity/complications [dehydration, sepsis, bronchopneumonia and/or others] appeared during the period of admission were managed.

### Data collection procedure

The sources of data were the inpatient hospital records and checklists that were developed according to the standard treatment protocol for the management of SAM. Information collected were patient-related data, anthropometric measurements, comorbidities, type of SAM, treatment lines and others.

For the anthropometric measurements: in light clothing, young children < 2 years old were weighed on a sensitive baby and children > 2 years old were weighted on digital electronic scales scale [Health o meter scales]. The weight records were taken to the nearest 0.1 kg. The length of children < 2 years of age was measured in the recumbent position using wooden length board (Infantometer) [Seca 417]; while the standing height was measured for children aged 2 years or older by a stadiometer. The records were taken to the nearest 0.1 cm. Age and measurements of weight and height were plotted on the WHO and Z-score Growth Charts to determine the percentiles for each parameter. The anthropometric z-scores were calculated using the WHO 2006 children growth references [21] which were computed based on the observation difference from the median values rather than the mean. These data were collected at the time of hospital admission, during hospitalization, at discharge and at all available post-discharge follow-up appointments up to 24 weeks.

During hospital stay, children’ parents/guardians were interviewed using a detailed structured questionnaire which inquired about the socioeconomic status and contact details (phone and address). Fahmy and El-Sherbini’s Social Classification Scale for assessing Egyptian socioeconomic status was used to classify the family socioeconomic status. This scale encompasses variables representing paternal education and work, housing conditions and family size and per-capita monthly income. Scores of 25–30 were considered a high social status, 20- < 25 were regarded as middle social status, 15 to < 20 indicated low social status while very low social status was defined at scores < 15; details were given elsewhere [22].

Children were discharged from the hospital not on the basis of specific anthropometric measurement [14], but after achieving the following WHO criteria: a well and alert child with good appetite and without medical complications including resolving of edema [1, 3].

### Follow-up procedure

All of the enrolled children who have survived hospitalization were requested to attend follow-up appointments for 6 months post-discharge as per routine follow-up schedule for the Nutrition Rehabilitation Unit in the hospital. Follow-ups were planned weekly for the first 2 weeks following discharge and biweekly thereafter. Routine procedures in each follow-up appointment included taking anthropometric measurements and vital signs, as well as assessing and managing of any current illness.

The main study outcome was to estimate the total, in-hospital and post- discharge survival status and mortality predictors among those under 5 children with SAM.

### Statistical analysis:\

Data entry and analyses were all done with IBM compatible computer using the SPSS for windows software version 22. Graphics were edited by the Excel Microsoft office 2013 software. Demographic and clinical characteristics at time of hospital admission for all admitted children and those died from SAM (total, in-hospital and post-discharge) were presented by mean and standard deviation for quantitative variables, while qualitative data were presented by frequency distribution.

Kaplan–Meier curves were plotted for the cumulative survival across the time of hospital stay (from hospital admission to hospital discharge in days), across the post-discharge period (from hospital discharge to the end of the post-discharge follow-up time in weeks) and across the total period between time of hospital admission to the end of follow-up appointments (in weeks).

Because the admission time was not fixed; some children were discharged earlier than others; we calculated the odds ratios (ORs) with its 95% confidence intervals (CIs) for the in-hospital deaths by the logistic regression analysis. While for total and pos-discharge mortality outcomes, the multivariable-adjusted hazard ratios (HRs); 95% confidence intervals (CIs) were calculated by the Cox proportional hazard regression. Both the logistic and Cox regression models were adjusted for sociodemographic and clinical variables at time of admission.

For the post-discharge and total mortality/survival analysis, children who started the follow-up plan and were not seen in subsequent appointments, with no knowledge of their death, were treated as censored cases at the time of their last follow-up appointment. Person-weeks of follow-up were calculated from time of hospital admission (for the total SAM mortality analysis) and from time of being discharged a live from the hospital (for the post-discharge mortality analysis) to one of the denouements outcomes (death, lost to follow-up or end of the study, i.e. complete 24 weeks of follow- up). There was no evidence of violation of the Cox proportional hazard assumptions as the *p*-value of the Schoenfeld residuals test were 0.71and 0.41 for models testing total and post-discharge SAM deaths, respectively. A statistically significant level was considered when a two-sided p-value was less than 0.05.

## Results

**Fig. 1:**
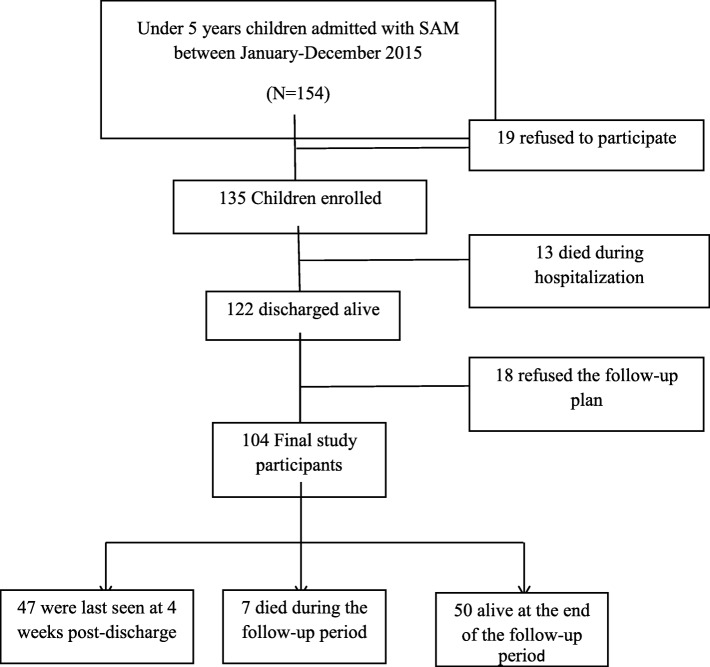
Flowchart of study subjects from admission until final follow-up. The downward arrows guide to the total number of children at the next step, and the sidebar lines guide to the number of excluded children or children with outcomes

The age range of all admitted children within the specified period of the study ranged from 6 to 59 months. We excluded 3 children who have died within 6 h of admission after revising their records and they were of extreme WHZ (− 5), height for age z scores (HAZ) of − 6 or weight for age z scores (WAZ) of − 6; however, we could not verify if these measurements were valid or were not correctly measured. Thus, a total of 154 children were hospitalized with SAM; however, a total of 19 caregivers were unwilling to participate, leaving 135 children eligible for the study, with a response rate 87.7%. An informed consent was taken from the children’s caregivers. During hospitalization, 13 children died (9.6% in-hospital death rate) and 122 (90.4%) were discharged alive with a follow-up plan. Parents of 18 discharged children refused to participate in the follow-up plan; leaving 104 children in the follow-up plan. Out of 104 alive discharged children and their parents consented to participate in the follow-up study, 7 cases have died (3, 2 and 2 death cases occurred at weeks 4, 6 and 8 post-discharge, respectively) and 47 other discharged children were last seen at 1 month after discharge, with no knowledge of outcomes. Therefore, in the survival analyses of the post-discharge and total SAM outcomes, we treated those lost to follow-up children as censored at last time seen (Fig. 1).

The mean age of the initially included 135 children was 10.2 ± 8.6 months, and 49.6% of them were males, 60% were rural residents and 57% belonged to families with very low/low socioeconomic status. Out of 135 children, 90 (66.7%) had edema, with or without a WHZ of <− 3 SD. The mean length of hospital stay was (15.47 ± 6.2) days, with the minimum and maximum lengths being 6 and 35 days, respectively. More than half of the admitted children (51.1%) had some co-morbidities/complications on admission in the form of dehydration (17.0%), bronchopneumonia (11.9%) and sepsis (9.6%) (Table 1).

**Table 1:** Demographic and admission characteristics for 135 severely malnourished children admitted to Minia University Hospital, January to December 2018

Variables	Number (%)
**Age (months)**	
6–11 m	98 (72.6)
12–24 m	31 (23.0)
> 24 m	6 (4.4)
**Sex**	
Males	67 (49.6)
Females	68 (50.4)
**Residence**	
Rural	81 (60.0)
Urban	54 (40.0)
**Socioeconomic score**	
Very low social standard	33 (24.4)
Low social standard	44 (32.6)
Middle social standard	48 (35.6)
High social standard	10 (7.4)
**Mother’s education**	
Educated	94 (70.0)
Illiterate	41 (30.0)
**Admission anthropometric characteristics**	
WAZ < −3SD without edema	9 (7)
Edema with WAZ < −3SD	51 (37.8)
Edema without WAZ < −3SD	49 (36.3)
Stunting (HAZ < −2SD)	8 (5.9)
Severe stunting (HAZ < −3SD)	1 (0.7)
Wasting (WHZ < −2SD)	8 (5.9)
Severe wasting (WHZ < −3 SD)	127 (94.1)
**Length of hospital stay**^**a**^	15.47 ± 6.2
**Co-morbidity/complication at admission**	
Dehydration	23 (17.0)
Bronchopneumonia	16 (11.9)
Sepsis	13 (9.6)
Others	6 (4.4)
More than one of the above complications	11 (8.1)

N.B. *WAZ* weight for age z-score; *HAZ* height for age z-score; *WHZ* weight for height (Z score)

^**a**^ data presented as mean **±** SD

Table 2 shows the demographic and clinical characteristics of total (20 cases), in-hospital (13 cases) and post-discharge (7 cases) children died from SAM. Most deaths (especially in-hospital deaths) occurred among younger age, rural children of low socioeconomic levels, and among those with the worse clinical presentations of WAZ, WHZ, edema and complications. Thirteen out of 135 SAM cases, admitted to the hospital, died within 4 weeks of admission; the overall during hospitalization death rate was 9.6%. The most common causes of in-hospital death were the malnutrition itself (8 cases), pneumonia (3 cases) and sepsis (2 cases). Whereas, 7 out of 104 discharged alive children died within 8 weeks after discharge making the post-discharge mortality rate = 6.7% and was mostly due to pneumonia in 6 cases and one case was reported to die from severe uncontrolled bleeding per orifices. The mean time from hospital admission till death were almost 13 days for 13 in-hospital deaths and 51 days for 7 post-discharge deaths. Out of total 122 children discharged alive, only 38 (31.1%) of them had achieved target weights of 85% weight for height at the time of discharge. The average weight gain was 10.4 g/kg/day (13 g/kg/day for children with severe wasting and 7.3 g/kg/day for children with edematous malnutrition) (Data not shown in tables).

**Table 2:** Demographic and clinical characteristics at time of hospital admission for children died from severe malnutrition (SAM)

	Total SAM deaths, n (%)	In-hospital deaths, n (%)	Post-discharge deaths, n (%)
**Age (months)**			
6–11 m	16 (80.0)	11 (84.6)	5 (71.4)
12–24 m	3 (15.0)	1 (7.7)	2 (28.6)
> 24 m	1 (5.0)	1 (7.7)	0 (0)
**Sex**			
Male	13 (65.0)	10 (76.9)	3 (42.9)
Female	7 (35.0)	3 (23.1)	4 (57.1)
**Residence**			
Rural	18 (90.0)	12 (92.3)	6 (85.7)
Urban	2 (10.0)	1 (7.7)	1 (14.3)
**Socioeconomic score**			
Low social standard	17 (85.0)	12 (92.3)	5 (71.4)
Middle social standard	3 (15.0)	1 (7.7)	2 (28.6)
High social standard	0 (0.0)	0 (0.0)	0 (0.0)
**Mother’s education**			
Illiterate	15 (75.0)	11 (84.6)	4 (57.1)
Educated	5 (25.0)	2 (15.49)	3 (42.9)
**WAZ**			
< −3SD	16 (80.0)	11 (84.6)	5 (71.4)
Not < −3SD	4 (20.0)	2 (15.4)	2 (28.6)
**WHZ**			
< − 3SD	19 (95.0)	13 (100.0)	6 (85.7)
Not < −3SD	1 (5.0)	0 (0.0)	1 (14.3)
**Edema**			
Edematous malnutrition	17 (85.0)	11 (84.6)	6 (85.7)
Non-edematous malnutrition	3 (15.0)	2 (15.4)	1 (14.3)
**Co-morbidity/complication at admission**			
Complicated^a^	16 (80.0)	10 (76.9)	6 (85.7)
No complications	4 (20.0)	3 (23.1)	1 (14.3)
**Length of hospital stay**^**b**^	14.1 ± 7.0	13.4 ± 6.1	15.3 ± 9.0
**Time from admission to death**^**b**^	26.5 ± 20.5	13.4 ± 6.1	50.9 ± 13.9

^a^SAM complicated with one or more of the following comorbidities dehydration, bronchopneumonia, sepsis and others

^**b**^Mean SD, all such variables

**Fig. 2:**
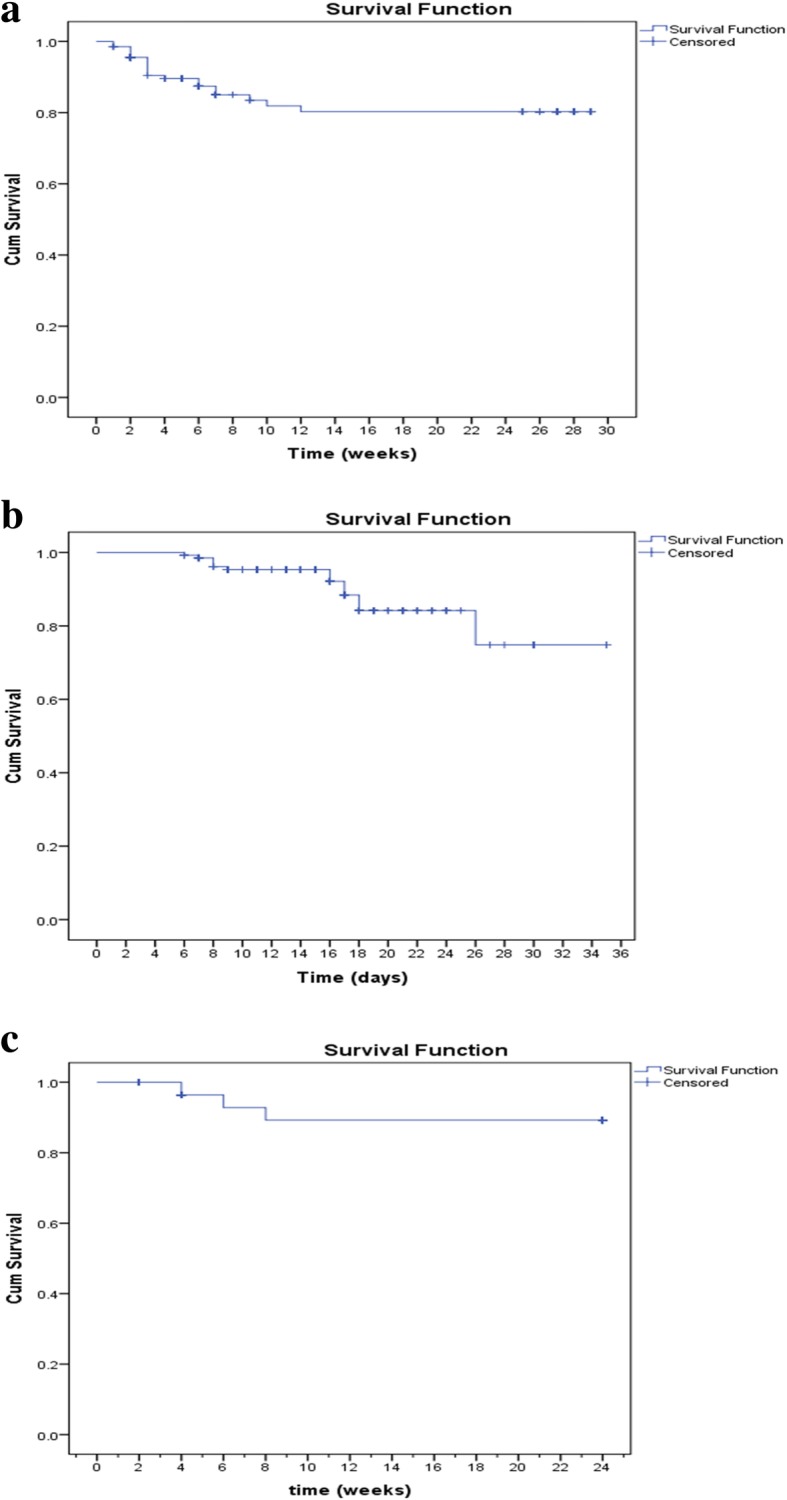
**a** Kaplan Meier curve for total deaths from admission until death or end of the study at 24 weeks post-discharge. The blue line represents the cumulative survival function across the time (from admission until death or end of the study at 24 weeks post-discharge) and the cross signs represent when data were censored at time of death (in-hospital or post-discharge) or last time seen. **II.b** Kaplan Meier curve for in-hospital deaths from admission until death or hospital discharge alive). The blue line represents the cumulative survival function across the hospital stay time and the cross signs represent when data were censored at time of in-hospital death. **II.c** Kaplan Meier survival curve for post-discharge mortality from SAM from time of hospital discharge until post-discharge death, lost to follow-up or end of follow at 24 weeks post-discharge. The blue line represents the cumulative survival function across the follow-up time and the cross signs represent when data were censored at time of post-discharge death or last time seen

The Kaplan–Meier curves for cumulative survival (Fig. 2. a,. b, and .c) show that 2 cases of in-hospital deaths occurred in the first week of admission, 4 cases in the second week, 6 cases in the third week of admission and only one case died at day 26 of the fourth week of admission. No in-hospital mortality occurred in the fifth week of admission. For the post-discharge mortality, all deaths had happened soon within 8 weeks after discharge; 3 cases at week 4, 2 cases at week 6 and 2 cases at week 8 post-discharge. For the total SAM outcome (in-hospital and post-discharge), 20 cases out of 135 children (14.8%) have died. The cumulative probability of a child to be discharged alive is 98.5% after being admitted with SAM for 1 week, 95.4% for 2 weeks admission, 87% for 3 weeks admission and 82.4% for 4 weeks or more admission. The cumulative probability for alive discharged child to survive up to 4 weeks after discharge was 96.4%, up to 6 weeks after discharge was 92.8%, and beyond 8 weeks and at least for 24 weeks after discharge was 89.3%. For under 5 children admitted with SAM, the cumulative probability of survival for 13 weeks (5 weeks maximum admission duration and 8 weeks early post-discharge follow-up) was 80.3%.

The logistic and Cox regression models included sociodemographic and clinical variables in a stepwise order showed that age, WAZ, edema and complications at admission to associate with both in-hospital and total SAM mortality. The adjusted ORs (95%CIs) for in-hospital SAM deaths were 2.64 (1.22–6.43) in children < 12 vs ≥ 12 months old, 8.10 (2.16–11.67) in those with WAZ < −3SD, 3.04 (1.70–6.06) in those with edema at admission and 3.71 (1.59–6.78) in children with complications. The respective HRs (95%CIs) for total SAM deaths according to the same groups of children were 1.57 (1.10–2.99), 4.79 (2.23–6.10), 2.99 (1.16–4.66) and 3.44 (1.07–9.86). Discharged children of illiterate mothers versus those of educated mother had a HR (95%CI) of post-discharge mortality of 7.10 (1.58–31.93) (Table 3).

**Table 3:** Multivariable regression* for factors associated with mortality (in-hospital, early post-discharge and total) from severe malnutrition (SAM) in children admitted to Minia University Maternity and Children Hospital, January to December 2018

	Cases/Total (n)	HR/OR (95%CI)**
**Total SAM deaths**	20/135	
***Age***		
6–12 m	16/98	1.57 (1.10–2.99)
> 12 m	4/37	Reference
***WAZ***		
Not < −3SD	4/75	Reference
< − 3SD	16/60	4.79 (2.23–6.10)
***Edema***		
Non-edematous malnutrition	3/45	Reference
Edematous malnutrition	17/90	2.99 (1.16–4.66)
***Co-morbidity/complication at admission***		
No complications	4/66	Reference
Complicated***	16/69	3.44 (1.07–9.86)
**In-hospital SAM deaths**	13/135	
***Age***		
6–12 m	11/98	2.64 (1.22–6.43)
> 12 m	2/37	Reference
***WAZ***		
Not < −3SD	2/75	Reference
< −3SD	11/60	8.10 (2.16–11.67)
***Edema***		
Non-edematous malnutrition	2/45	Reference
Edematous malnutrition	11/90	3.04 (1.70–6.06)
***Co-morbidity/complication at admission***		
No complications	3/66	Reference
Complicated***	10/69	3.71 (1.59–6.78)
**Early post-discharge SAM deaths**	7/104	
***Mother’s education***		
Educated	3/84	Reference
Illiterate	4/20	7.10 (1.18–31.98)

*The logistic regression analysis was used for in-hospital SAM deaths and the Cox regression analysis was used for total and post-discharge SAM deaths

**For both the logistic and Cox regressions, the stepwise multivariable models included age, sex, residence, socioeconomic status, mother’s education, WAZ, WHZ, edema and comorbidity/complication at admission

***SAM complicated with one or more of the following comorbidities dehydration, bronchopneumonia, sepsis and others

## Discussion

In this study, the cumulative probability of a child admitted with SAM to be discharged alive at the end of the fourth week of hospital admission was 82.4%. Age ≤ 12 months, WAZ < −3SD, presence of edema and complications at admission were independent predictors of both in-hospital and total SAM mortality; whereas, the mother’s education attributed to the early post-discharge mortality.

Over 70% of children hospitalized with SAM in our study were under 1 year of age. This confirms a high prevalence of SAM in younger children reported previously [23, 24]. We found age < 12 months was associated with total and in-hospital SAM deaths. This agreed with the findings of an Ethiopian study by Jarso et al. [10] which showed younger age children with SAM were two times more likely to die earlier. Younger children may be more vulnerable because of depressed immunity, increased risk of infection and insufficient feeding practices [10]. This finding was also supported by findings from other African studies [9, 25, 26].

Children with comorbidities/complication at admission were 3.25 times more likely to die than children without co-morbidities/complication in our study. Similar results were reported in Ethiopia by Jarso et al. (2015) and more recently by Guesh et al. (2018) [10, 26]. Complicated SAM is typically associated with an in-patient mortality risk of 12% to more than 30% in African hospitals [11, 27–29] A meta-analysis of inpatient treatment outcomes of SAM among under-5 children in Ethiopia concluded that comorbidities at admission were predictors of mortality [30].

Having edema at admission was associated with the in-hospital death in our study. Bachou et al. [31] who studied risk factors of the in-hospital death in children with SAM in Uganda found that the presence of edema increased the odds of death occurring in the first week of admission, but did not reach significance; OR (95% CI) was 2.0 (0.8–4.7). To the contrary, Gebremichael et al. reported that Ethiopian SAM’s children who were diagnosed as edematous malnourished were more likely to recover earlier than their severe wasting counterparts [32]. Also, Gachau et al. reported that edema was not associated with the increased mortality among Kenyan children hospitalized with SAM [33]**.**

In this follow-up study for children with SAM, being a child of an educated mother was a significant predictor for a long-term survival in post-discharged SAM children admitted to and discharged alive from the Nutrition Rehabilitation Unit in Minia University Maternity and Children Hospital and completed 6 months of follow-up with a post-discharge mortality rate = 6.7%. A study conducted among Bangladeshi under-5 children reported that 3-month post-discharge mortality rate following hospitalization with SAM and pneumonia was 8.7% [20]. Another study that included 393 Malawian children with SAM reported an 11% mortality rate within 3 months of hospital discharge [13]. The discrepancies in the reported mortality rates in the previous studies may be related to different study inclusion criteria, hospital discharge criteria or study populations characteristics (for example the level of mother’s education as indicated by our findings). Several previous studies had shown the mothers’ education one of the main determinants of under-5 mortality [34–37]. Educated mothers versus illiterates are logically capable of coping with not only skills needed in post-discharge healthcare practices and disease treatment, but also those related to preventive care, such as child hygiene and nutrition, thus improving chances for the child survival [34–36, 38].

An important consideration is the timing of the post-discharge deaths, most deaths had happened during the first several weeks of discharge, which indicates the importance of making the intervention during this period to help reduce the burden of deaths. These early deaths would suggest a continuation of the acute illness not completely recovered during admission period. Thus, mother’s education could attribute to SAM’s mortality that is attributable to acute illness episode and manifests beyond inpatient treatment.

Strengths of this study include that data regarding the mortality predictors were collected at admission, before the discharge decision was made or the post-discharge outcomes were known, which reduced the potential for selection bias.

### Limitations of the study

First, the reliability of the recorded data could not be ascertained. Our data did not register the MUAC for all admitted children (there were 42 missed cases, among the recorded cases; 37 children had MUAC< 11.5 cm) thus some selection bias was unavoidable. Second, according to the rule of thumb in statistics, Cox regression models should have at least 10 outcomes for one independent variable. Although this was fulfilled for models assessed the total and in-hospital SAM deaths; however, only 7 cases were confirmed as post-discharge SAM mortalities. Moreover, the proportion of children who were lost after 1 month of post-discharge follow up is huge; 45% of children assigned for the follow-up plan. Therefore, the findings of the Cox model for the post-discharge mortality should be considered with caution regarding its validity. The confirmation of the study findings, especially for the post-discharge mortality, by further large-scale studies, is needed.

## Conclusion

This retrospective cohort study showed that, during hospitalization, the death rate of children with SAM admitted to the Nutrition Rehabilitation Unit in Minia University Maternity and Children Hospital reached the SPHERE target of < 10% [37]. Younger age and the SAM presented with WAZ- > 3 SD, edema or complication at admission were significant predictors of both total and in-hospital mortality from SAM. Mothers’ illiteracy was shown to be associated with the post-discharge deaths which occurred early within 8 weeks of hospital discharge. The results of our study indicate that the early post-discharge care represents a crucial integral extension of the hospital management for children with SAM. We recommend nutritional and hygiene educational programs for the caregivers of children with SAM during hospital admission and at time of discharge with emphasizing on the need for continued access to health-care facilities and interventions to reduce the acquisition of new infections and to receive treatment for such conditions.

## Data Availability

The datasets generated and/or analyzed during the current study are not publicly available due [data contained from hospital records] but are available from the corresponding author on reasonable request.

## Abbreviations

SAM: Severe Acute Malnutrition
WFL/WFH: weight for length/height
